# MMP3-Mediated tumor progression is controlled transcriptionally by a novel IRF8-MMP3 interaction

**DOI:** 10.18632/oncotarget.3897

**Published:** 2015-05-08

**Authors:** Debarati Banik, Colleen S. Netherby, Paul N. Bogner, Scott I. Abrams

**Affiliations:** ^1^ Department of Immunology, Roswell Park Cancer Institute, Elm and Carlton Streets, Buffalo, NY 14263, USA; ^2^ Department of Pathology, Roswell Park Cancer Institute, Elm and Carlton Streets, Buffalo, NY 14263, USA

**Keywords:** host-tumor interaction, interferon regulatory factor-8, matrix metalloproteinase-3, metastasis, tumor progression

## Abstract

Interferon regulatory factor-8 (IRF8), originally identified as a leukemic tumor suppressor, can also exert anti-neoplastic activities in solid tumors. We previously showed that IRF8-loss enhanced tumor growth, which was accompanied by reduced tumor-cell susceptibility to apoptosis. However, the impact of IRF8 expression on tumor growth could not be explained solely by its effects on regulating apoptotic response. Exploratory gene expression profiling further revealed an inverse relationship between IRF8 and MMP3 expression, implying additional intrinsic mechanisms by which IRF8 modulated neoplastic behavior. Although MMP3 expression was originally linked to tumor initiation, the role of MMP3 beyond this stage has remained unclear. Therefore, we hypothesized that MMP3 governed later stages of disease, including progression to metastasis, and did so through a novel IRF8-MMP3 axis. Altogether, we showed an inverse mechanistic relationship between IRF8 and MMP3 expression in tumor progression. Importantly, the growth advantage due to IRF8-loss was significantly compromised after silencing MMP3 expression. Moreover, MMP3-loss reduced spontaneous lung metastasis in an orthotopic mouse model of mammary carcinoma. MMP3 acted, in part, in a cell-intrinsic manner and served as a direct transcriptional target of IRF8. Thus, we identified a novel role of an IRF8-MMP3 axis in tumor progression, which unveils new therapeutic opportunities.

## INTRODUCTION

Increased resistance of neoplastic subpopulations to cell death is well-regarded as a major hallmark of tumor progression [1]. Therefore, understanding the molecular bases for tumor-cell resistance to cell death is critically important not only to improve our knowledge of cancer biology, but also to develop more effective cancer therapies. Prior work in our laboratory identified a previously undescribed role for the transcription factor, interferon regulatory factor-8 (IRF8) in the mechanism of death receptor-mediated killing of solid malignancies. We [2–4] and others [5, 6] showed that IRF8 augments Fas-mediated cell death, and does so by inhibiting the expression of several key anti-apoptotic proteins of the Fas pathway, Bcl-xL, PTPN13 or FLIP.

IRF8 was originally discovered as a transcription factor essential for regulating normal myelopoiesis [7–9]. Intriguingly, loss of IRF8 within the myeloid compartment results in a myeloproliferative disorder due, in part, to a decrease in apoptotic sensitivity. Over time, this myeloproliferative phenotype transitions to a chronic myeloid leukemia (CML)-like syndrome in a significant fraction of IRF8-null mice [7]. In humans, substantially reduced IRF8 mRNA levels have been reported in patients with CML or acute myeloid leukemia (AML) [10–12]. Thus, IRF8 was initially implicated as a ‘tumor suppressor gene’ in certain hematopoietic cancers [7–13]. Furthermore, we [14] and others have now found that IRF8 can be expressed, but typically is epigenetically silenced in non-hematopoietic malignancies [15, 16] or repressed by immune-associated soluble factors during tumor-induced alterations in myelopoiesis [17]. Re-expression of IRF8 by epigenetic or molecular approaches significantly restores apoptotic responsiveness and reduces aggressive behavior *in vitro* or *in vivo* including primary and metastatic tumor growth [2, 15, 18–20]. While IRF8 is recognized as a tumor suppressor gene in certain hematopoietic malignancies [7–13], in solid tumor models, these additional studies [2, 3, 15, 18–20] underscore previously unrecognized anti-neoplastic activities for IRF8 perhaps distinct from its tumor suppressor roles.

Loss-of-function experiments confirmed a causal role for IRF8 expression in regulating tumor growth *in vivo* [3]. In those studies, we made use of the mouse CMS4 tumor model [21], which enabled us to experimentally manipulate endogenous IRF8 levels by RNA interference. We found that IRF8-loss significantly enhanced tumor growth rate compared to the vector control cells [3]. Interestingly, increases in tumor growth *in vivo* could not be explained solely by differences in apoptotic phenotype [3], suggesting that additional aspects of tumor biology were influenced by IRF8 expression. Consequently, to gain broader insights into the molecular basis for this IRF8-dependent tumor growth advantage, we performed preliminary microarray studies using the IRF8-expressing/IRF8-deficient CMS4 mouse isogenic tumor pair. In doing so, we identified an unrecognized inverse relationship between IRF8 and MMP3 expression, but not with other members of the matrix metalloproteinase (MMP) family.

Generally, MMPs mediate a spectrum of enzymatic activities that profoundly alter tissue architecture under both physiologic and pathologic conditions, including neoplasia [22–24]. While MMPs, namely MMP2 [25] and MMP9 [26] have been well-studied in cancer biology, less is known about the role of MMP3. MMP3 belongs to the Stromelysin family of MMPs and has exhibited broad substrate specificity, making it a critical player in extracellular matrix remodeling. Its role in cancer biology, however, was first recognized when enforced MMP3 expression in murine mammary gland epithelium led to early tumorigenesis [27]. Ectopic expression of MMP3 [28, 29] or the addition of recombinant MMP3 protein [26] was shown to activate Rac1b-dependent pathways, culminating into genomic instability and subsequent acquisition of an epithelial-to-mesenchymal transition [28, 29] or increased invasion and activation of heightened malignant transcriptional profiles [26]. An indirect mechanism for MMP3-mediated tumorigenesis is thought to involve cleavage of E-cadherin and subsequent activation of the β-catenin pathway [28].

Moreover, recent work by Bissell, Werb and colleagues demonstrated a functional role for MMP3 during hypermorphic epithelial outgrowth via effects on mammary stem cells, which reinforces the relevance of MMP3 during tumor initiation/promotion [30, 31]. In humans, polymorphisms within the MMP3 promoter region have carried prognostic merit. For example, the 5A vs. 6A single nucleotide polymorphism at position −1171 upstream from the transcription start site has been associated with over-activation of MMP3 promoter activity and higher incidences of cancer [32]. How MMP3 is transcriptionally regulated, particularly in cancer models, has also remained less understood. While it is known that growth factor/cytokine-mediated induction of MMP3 expression involves signaling through AP-1 [33] and ETS [34] or interaction of both families of transcription factors [35], limited data are available regarding transcriptional mechanisms that oversee MMP3 downregulation. Based on earlier findings that IRF8-loss augmented tumor growth [3, 18] and that this malignant phenotype was inversely associated with an unexpected increase in MMP3 expression (by microarrary analysis), we hypothesized that MMP3 is downregulated by a novel IRF8-dependent mechanism. We further hypothesized that MMP3 influences the neoplastic process not only at the stage of early tumorigenesis as originally reported [27], but also at later stages of tumor progression to metastatic disease. Altogether, in several mouse tumor models, we describe a novel transcriptional mechanism of MMP3 regulation by IRF8, and showed that MMP3 expression plays an underappreciated and key role in later stages of tumor progression, including metastasis.

## RESULTS

### IRF8 is a negative regulator of MMP3 expression and function

**Figure 1:**
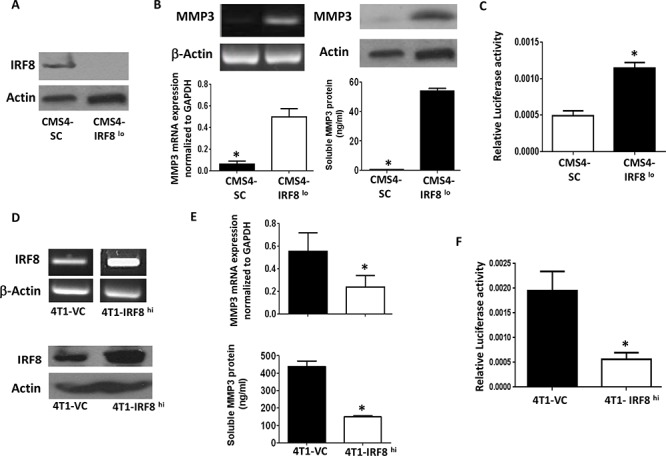
Inverse causal relationship between IRF8 and MMP3 expression **A.** Knockdown of IRF8 expression in the parental CMS4 cell model; scrambled control (SC) vs. CMS4-IRF8^lo^ cells, as shown by Western blot. **B.** MMP3 mRNA levels (RT-PCR, upper left; qPCR) or MMP3 protein levels (Western blot, upper right; ELISA, lower right) measured in the same populations as in *A*. **C.** Relative MMP3 promoter activity in vector control vs. CMS4-IRF8^lo^ cells, as measured by dual luciferase assay. **D.** IRF8 expression in 4T1 cells after transfection with an expression plasmid encoding the murine IRF8 gene or an empty vector control (VC). RT-PCR (upper panel) or Western blot (lower panel). **E.** Effect of enforced IRF8 expression on MMP3 levels as measured by qPCR (upper panel) or ELISA (lower panel). **F.** Relative MMP3 promoter activity in VC vs. 4T1-IRF8^hi^ cells, as in *C*.

To extend our preliminary observations from the gene expression data, we examined MMP3 expression at multiple molecular and biochemical levels, first in the same CMS4 tumor model. Here, we made use of a previously established cell line system whereby basal IRF8 expression levels were altered by RNA interference [3]. Consistent with what we observed at the mRNA level [3], we showed that IRF8-deficient CMS4 cells (CMS4-IRF8^lo^) expressed substantially reduced levels of IRF8 protein compared to the scrambled control population (CMS4-SC) (Fig. 1A). Next, the inverse relationship between IRF8 and MMP3 expression was confirmed at both RNA (RT-PCR and real-time RT-PCR, Fig. 1B; left upper and lower panels) and protein (Western blot and ELISA, Fig. 1B; right upper and lower panels) levels. Moreover, using a previously designed MMP3 luciferase-reporter vector [36, 37], we showed that transient transfection of CMS4-IRF8^lo^ cells led to enhanced MMP3 promoter activity compared to CMS4-SC cells (Fig. 1C), indicating that IRF8 acted as a negative regulator of MMP3 expression.

The nature of this inverse IRF8-MMP3 relationship was then investigated in a second tumor model. Here, we made use of an IRF8 gain-of-function approach using 4T1 cells (Fig. 1D), since the parental 4T1 cell line (data not shown) or the empty vector control (Fig. 1E) expressed high basal levels of MMP3. Overexpression of IRF8 in 4T1 cells (4T1-IRF8^hi^; Fig. 1D; RT-PCR and Western Blot) resulted in diminished MMP3 expression at both RNA (real-time RT-PCR) and protein (ELISA) levels (Fig. 1E) compared to the 4T1 vector control cells (4T1-VC). We also observed a concomitant decrease in MMP3 promoter activity in 4T1-IRF8^hi^ cells (Fig. 1F). Together with our CMS4 experiments, these data supported the hypothesis that IRF8 negatively regulated MMP3 transcription either directly or indirectly.

**Figure 2:**
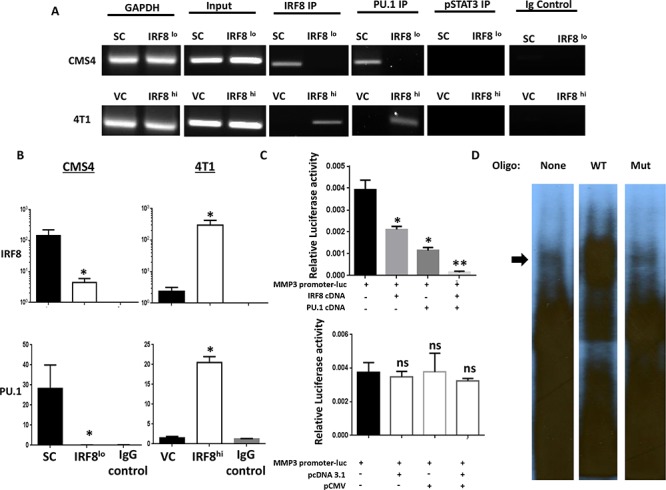
IRF8 regulates MMP3 promoter activity in conjunction with PU.1 **A.** Chromatin immunoprecipitation (ChIP) followed by RT-PCR on the indicated cell lines to determine binding of IRF8 and PU.1 to the putative consensus motif of the mouse MMP3 promoter (−1137 base pairs upstream of the TSS). Data represent one of two independent experiments. **B.** ChIP followed by quantitative PCR on the indicated cell line to determine enrichment of IRF8 or PU.1 at the putative consensus motif. **C.** Transfection assays using the CMS4 cell line to measure MMP3 promoter activity in the presence of cDNA encoding full-length IRF8, PU.1 cDNA or both (upper panel) vs. the corresponding empty vector control plasmids (lower panel). **D.** EMSA using CMS4-SC lysates after incubation with or without the indicated ^32^P-labeled oligonucleotide probe. Data are representative of two separate experiments.

To explore the possibility for a direct binding interaction between IRF8 and elements of the MMP3 promoter, we first visually inspected the murine MMP3 promoter *in silico* for IRF8 binding sites. In doing so, we identified a putative IRF8 binding site reflecting both ISRE (interferon-stimulated response element) and EICE (Ets/IRF composite element) motif characteristics [38] starting at position −1137 upstream from the transcriptional start site (GGAATGGAAA; Suppl. Fig. 1). ChIP assays were then performed using the IRF8-expressing or IRF8-deficient CMS4 cells. The protein-DNA complex was incubated with a ChIP-certified anti-IRF8 antibody, followed by PCR amplification of the MMP3 promoter region using primers surrounding a putative ISRE site located −1137 bp upstream from the transcription start site (Suppl. Fig. 1). Using the CMS4-SC cells, we found a direct binding interaction between IRF8 and the MMP3 promoter, based on the appearance of a PCR product reflecting the expected fragment size and subsequent quantification of these data which revealed a significant increase in the ChIP signal relative to the input DNA (Fig. 2A and 2B). Specificity for this IRF8-MMP3 axis was shown in two ways. First, no PCR product was detectable using the isotype control antibody or an antibody reactive against an unrelated transcription factor (i.e., pSTAT3) and, secondly, little to no PCR product was detectable in CMS4-IRF8^lo^ cells (Fig. 2A). ChIP experiments were then performed using the 4T1 system and, as with the CMS4 system, a direct binding interaction was shown using 4T1-IRF8^hi^ cells (Fig. 2A and 2B). An additional control included a ChIP-PCR reaction for an unrelated genomic region (i.e., GAPDH), which supported the integrity of the input DNA in these preparations.

Because the transcriptional activity of IRF8 is typically enhanced when IRF8 partners with other transcription factors, namely PU.1 [38, 39], we then sought to determine whether this could be the case for MMP3 expression. Therefore, to test this hypothesis, we made use of both ChIP and reporter assays (Fig. 2 and Suppl. Fig. 2). First, we found that immunoprecipitation with anti-PU.1 antibody captured a DNA sequence consistent with what we observed for IRF8. This was observed both by image analysis and quantification of the ChIP signal (Fig. 2A and 2B). As with the IRF8 analysis, specificity was revealed using either an isotype control antibody or an antibody directed against an unrelated transcription factor (i.e., pSTAT3) that failed to capture the expected PCR product. Moreover, we sequenced the DNA fragment captured by immunoprecipitation with either the anti-IRF8 or anti-PU.1 antibody and confirmed that this particular DNA sequence in both cases contained the putative IRF8 binding motif (sequence underlined and marked in red in Suppl. Fig. 1).

Secondly, we modified the reporter assay in Fig. 1F to measure the impact of excess exogenous IRF8 and/or PU.l cDNA to abrogate MMP3 promoter activity (Fig. 2C). To do so, CMS4-SC cells were transiently transfected with the MMP3 promoter-luciferase construct alone or in combination with full-length expression plasmids for IRF8, PU.l or both. Our data indicated that the inclusion of expression plasmids for each transcription factor alone and even more so in combination significantly decreased MMP3 promoter activity (Fig. 2C, upper panel). In contrast, transfection of CMS4-SC cells with the respective empty vector control plasmids did not attenuate luciferase response, demonstrating specificity for IRF8- and/or PU.l-mediated inhibition of MMP3 promoter activity. Altogether, these data (Fig. 2 and Suppl. Fig. 2) are consistent with an IRF8-PU.1 partnership, which negatively regulates MMP3 promoter activity.

Lastly, we performed EMSA experiments using CMS4-SC cells as an *in vitro* model to investigate the IRF8-MMP3 interaction, making use a DNA construct reflecting the predicted IRF8 binding motif within the MMP3 promoter (Fig. 2D). To that end, we synthesized two types of oligonucleotides, one reflecting the wild-type (WT) sequence and one reflecting a mutant version containing insertional bases at both 5′ and 3′ ends of the motif thereby disrupting the potential DNA-protein interaction. Compared to control lysates incubated without any probe, cell lysates incubated with the WT probe showed effective retardation (Fig. 2D; upper region of the gel marked by arrow), indicative of a productive interaction. In contrast, cell lysates incubated with the mutant probe failed to show retardation and, in fact, the migration pattern mimicked that seen in the absence of any probe, unveiling specificity of the interaction.

Intriguingly, although 4T1 cells expressed basal levels of IRF8 (Fig. 1D), we did not observe a detectable binding interaction of IRF8 to the MMP3 promoter (Fig. 2A, lower panel). This observation suggested that endogenous IRF8 expression in 4T1 cells, while detectable by this antibody, may not be functional for yet unclear reasons, such as inappropriate accumulation within the cytoplasm rather than the nucleus. In an effort to explore this possibility, we made use of Image Stream technology, which enabled us to measure/confirm not only total IRF8 protein levels, but its cellular localization (Suppl. Fig. 3). Both human monocytic (THP-1 cells) and mouse macrophage (RAW264.7, henceforth ‘RAW’) cell line models were included as positive controls for IRF8 expression [17, 40]. To validate specificity of the assay, we included an IRF8-peptide competition step to block antibody binding [17].

First, we confirmed that the four cell lines tested expressed IRF8 but that the expression levels varied. This was based on two calculations, one being the difference in MFI values of the paired samples (i.e., absence vs. presence of the blocking peptide), and the other being the ‘D-value’, a nonparametric test to measure the difference between the two sample distributions ranging from 0 (i.e., no difference) to 1 (i.e., maximum difference). Based on these analyses, THP-1 cells had the highest expression level, while RAW, CMS4, and 4T1 cells seemed to express similar amounts of total IRF8 protein. Next, with respect to the compartmentalization of IRF8, THP-1 cells were found to express very strong nuclear staining that was abrogated by peptide competition. This was determined by the similarity score, which quantified changes in the overlap of IRF8 with DAPI (a nuclear dye). The similarity score is illustrated by the ‘Rd-value’, based on the Fisher's Discriminant Ratio, which considers the difference between the two distributions (i.e., absence vs. presence of the blocking peptide). As with THP-1 cells, strong nuclear IRF8 staining was observed with RAW cells. In contrast to THP-1 and RAW cells, CMS4 and 4T1 cells expressed lower levels of nuclear IRF8, which was evident by a reduced effect of the peptide to alter the Rd-value. The relatively high Rd-value of RAW cells compared to 4T1 and CMS4 cells combined with no difference in D-value among these 3 mouse cell lines is indicative for a relatively higher nuclear localization of IRF8 in RAW cells.

Altogether, these data demonstrated that while both CMS4 and 4T1 cells expressed comparably low levels of nuclear IRF8, it appeared unlikely that the IRF8-MMP3 interaction found in CMS4 cells but not 4T1 cells was due to cell-specific differences in the compartmentalization of endogenous IRF8. Therefore, the reasons for why CMS4 cells, but not 4T1 cells exhibit an IRF8-MMP3 interaction are likely due to other complex possibilities, such as cell-specific differences in the binding stability of nuclear IRF8 to the MMP3 promoter region which could impact the half-life of the protein-DNA interaction [41], defects in the IRF-association domain (IAD) of IRF8 which may alter the efficiency of transcription factor partnerships[42], or yet other unknown mechanisms.

### *In vivo* consequences of altering the IRF8-MMP3 axis

**Figure 3:**
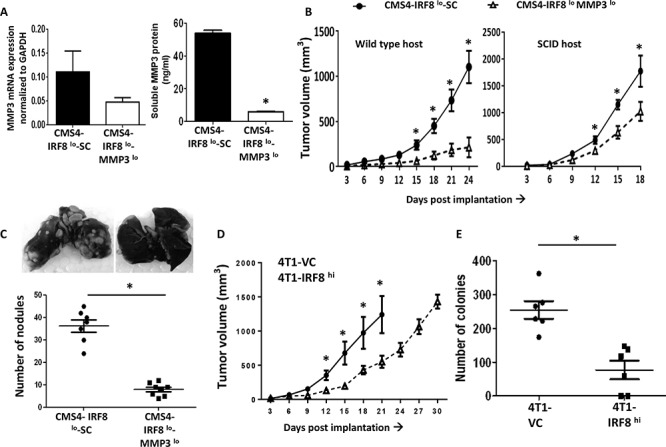
IRF8 regulates tumor behavior in an MMP3-dependent manner **A.** Knockdown of MMP3 expression in CMS4-IRF8^lo^ cells compared to the SC population; data confirmed at RNA (qPCR, left) and protein (ELISA, right panel) levels. **B.** Tumor growth in wild-type mice (*n* = 16 per tumor cell line) or SCID mice (*n* = 5 per tumor cell line). **C.** Quantification of experimental lung nodules of the indicated tumor cell line following India ink staining. Each data point represents a single mouse. Representative lung-stained images are shown from each group. **D.** 4T1-IRF8^hi^ or VC cells were orthotopically implanted into female wild-type BALB/c mice and tumor growth measured (*n* = 10 per tumor cell line). **E.** Lung-infiltrating tumor cells, assessed by clonogenic assays, using lung tissues from mice with similar primary tumor volumes (~1000 mm^3^). Each data point represents a single mouse. *P < 0.05.

Next, we examined whether manipulation of this IRF8-MMP3 interaction altered tumor growth *in vivo*. The approach taken was to silence MMP3 expression in the CMS4-IRF8^lo^ cells. The prediction was that if the enhanced rate of tumor growth observed with CMS4-IRF8^lo^ cells was due at least in part to higher MMP3 levels, then attenuating MMP3 expression altogether should reduce this IRF8-dependent growth advantage. Initial experiments analyzed knockdown efficiency of three separate shRNA-MMP3 constructs in the parental CMS4 cell line and identified two clones (clones #1 and #2) capable of significant gene-mediated silencing compared to the SC population (Suppl. Fig. 4). Based on knockdown efficiency, subsequent experiments were carried out with clone #1. Indeed, we found that decreased MMP3 expression in CMS4-IRF8^lo^ cells (Fig. 3A; at RNA and protein levels) led to a significant reduction not only in local tumor growth, but also experimental lung metastasis (Fig. 3B & 3C; Suppl. Fig. 5 for tumor growth assay designs). The observation that CMS4-IRF8^lo^ SC cells still grew faster than CMS4-IRF8^lo^MMP3^lo^ cells in SCID mice (Fig. 3B), which lack T and B cells, suggested that such differential tumor growth patterns can still occur without potential negative contributions of adaptive immune responses.

Conversely, 4T1-IRF8^hi^ cells grew significantly slower than the 4T1-VC population (Fig. 3D). Moreover, we observed a significant decline in 4T1 spontaneous lung metastasis even when the primary tumor remained intact, as measured by clonogenic assays using lung tissue from mice with similar primary tumor volumes (Fig. 3E). It is important to note that altering IRF8 or MMP3 levels did not alter *in vitro* proliferation or cell surface expression of MHC class I (e.g., H-2K^d^) (Suppl. Fig. 6) compared to the appropriate SC populations. Thus, these data revealed a previously undescribed IRF8-MMP3 axis in tumor biology.

### Impact of MMP3 expression on tumor growth and progression

**Figure 4:**
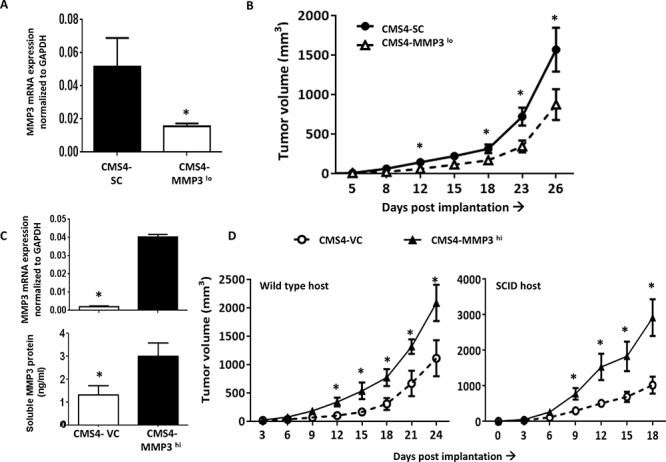
Modulating tumor-derived MMP3 levels alters CMS4 tumor growth **A.** MMP3 expression in parental CMS4 cells after stable MMP3 knockdown vs. SC, as shown by qPCR. **B.** Tumor growth illustrated for the indicated cell line (*n* = 16 mice per tumor cell line). **C.** Ectopic overexpression of MMP3 in CMS4 cells as shown at RNA (qPCR, upper panel) or protein (ELISA, lower panel) levels. **D.** Tumor growth in wild-type mice (*n* = 15 per tumor cell line) or SCID mice (*n* = 5 per tumor cell line). *P < 0.05.

Next, we sought to molecularly alter tumor-derived MMP3 levels in order to specifically focus on the role of MMP3 in tumor progression. Although MMP3 levels have been originally linked to tumor initiation/promotion [27], less is known about their role in subsequent stages of the neoplastic process. Initially, we selected CMS4 cells and either stably silenced (CMS4-MMP3^lo^) or overexpressed (CMS4-MMP3^hi^) the mouse MMP3 gene. Despite the fact that CMS4 cells expressed low basal levels of MMP3, silencing MMP3 further decreased tumor growth (Fig. 4A & 4B). Conversely, overexpressing MMP3 (Fig. 4C, at RNA and protein levels) enhanced *in vivo* tumor growth (Fig. 4D; Suppl. Fig. 4). Again, this effect was observed in both wild-type and SCID mice (Fig. 4D), and modifying MMP3 levels did not alter *in vitro* proliferation or MHC class I expression (e.g., H-2K^d^; Suppl. Fig. 6).

**Figure 5:**
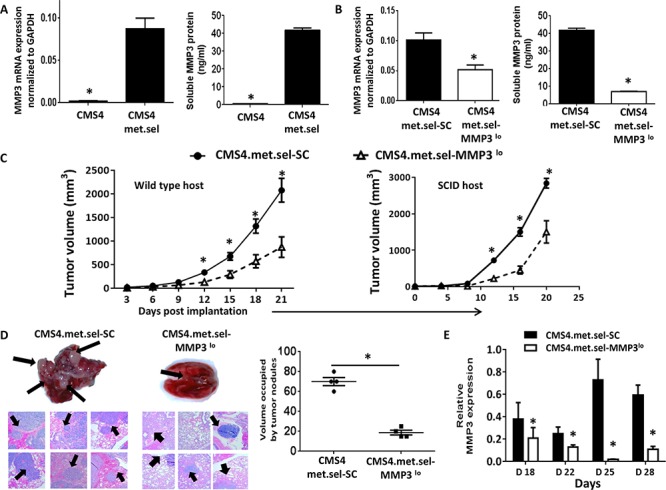
Modulating tumor-derived MMP3 levels alters CMS4-met.sel tumor growth **A.** Comparison of basal MMP3 expression between CMS4 and CMS4.met.sel at RNA (qPCR, left panel) or protein (ELISA, right panel) levels. **B.** Knockdown of MMP3 in CMS4.met.sel cells compared to the SC population as shown at RNA (qPCR, left panel) or protein (ELISA, right panel) levels. **C.** Tumor growth in wild-type mice (*n* = 18 per tumor cell line; left) or SCID mice (*n* = 5 per tumor cell line; right). **D.** Experimental lung metastases as shown by H&E-stained lung tissue sections (left), followed by quantification of approximate volume of lung occupied by tumor (right). Arrows indicate examples of lung tumor nodules. **E.** MMP3 levels in tumor explants at different time points post-implantation, as measured by qPCR. *P < 0.05 (experimental replicates).

Previously, we reported on the isolation of an aggressive *in vivo* variant of CMS4 (termed CMS4.met.sel), which expressed low basal levels of IRF8 [43]. Here, we found that such cells also expressed high levels of MMP3 compared to the parental cells (Fig. 5A, based on RNA and protein levels). When MMP3 expression was silenced in this CMS4 variant (Fig. 5B, based on RNA and protein levels), we observed significant decreases in tumor growth in both wild-type and SCID mice (Fig. 5C; Suppl. Fig. 5), as with CMS4-IRF8^lo^ cells (Fig. 3B). Similarly, we observed significant reductions in experimental lung metastases (Fig. 5D; Suppl. Fig. 5). In a separate cohort of mice, we collected tumor explants and verified knockdown of MMP3 expression during the course of tumor growth (Fig. 5E). Consistent with earlier findings, modifying MMP3 levels did not alter *in vitro* proliferation or MHC class I expression (e.g., H-2K^d^; Suppl. Fig. 6).

**Figure 6:**
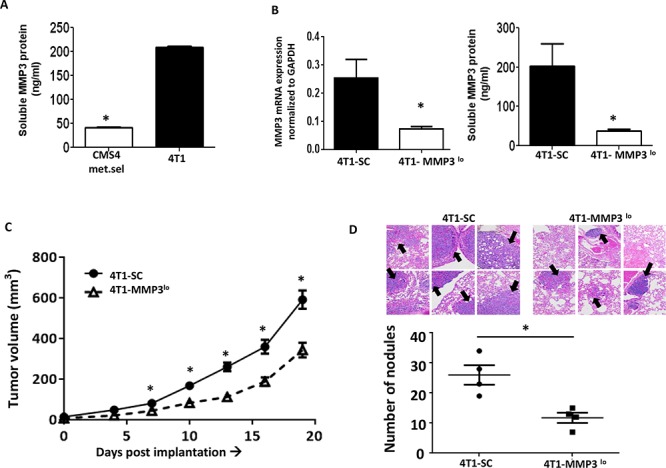
Modulating tumor-derived MMP3 levels alters 4T1 tumor growth **A.** Endogenous MMP3 production levels in 4T1 cells compared to CMS4.met.sel cells, as shown by ELISA. **B.** Knockdown of MMP3 in 4T1 cells compared to SC population as shown at RNA (qPCR, left panel) or protein (ELISA, right panel) levels. **C.** Tumor growth in wild-type mice (*n* = 18 for each tumor cell line). **D.** Experimental lung metastases as shown by H&E-stained lung tissue sections (upper) followed by histologic quantification of tumor nodules (lower). Arrows indicate examples of lung tumor nodules (*n* = 4 per group). *P < 0.05.

**Figure 7:**
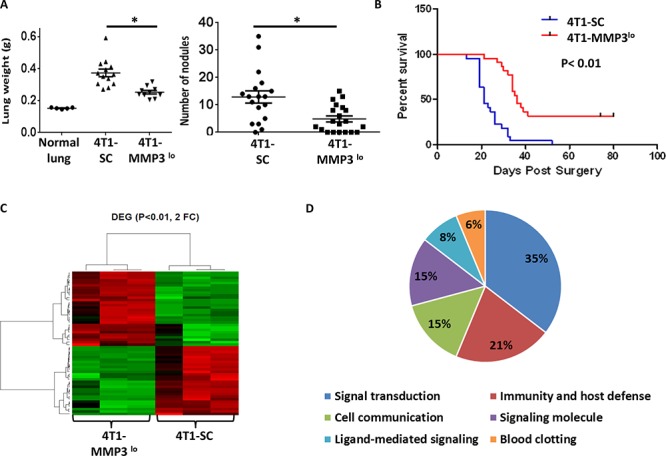
Tumor-derived MMP3 impacts spontaneous metastasis **A.** Quantification of spontaneous lung metastasis of the indicated 4T1 cell population with primary tumors left intact. Data collected at endpoint when mice harbored similar primary tumor volumes (4T1-SC: 1794 ± 147 mm^3^; 4T1-MMP3^lo^: 1798 ± 143 mm^3^) and quantified by lung weight or lesion count of H&E-stained lung tissue sections. Each data point represents a single mouse. **B.** After surgical removal of the primary tumor (i.e., 4T1-SC: 113 ± 9.2 mm^3^, 4T1-MMP3^lo^: 126 ± 5.5 mm^3^), mice were followed for survival based on the appearance of signs/symptoms of morbidity (*n* = 21 mice per tumor cell line). **C.** Gene expression profiling of the indicated 4T1 tumor cell population in biologic triplicates, as shown by heat-map of differentially expressed genes (*n* = 58; > 2-fold up or down; *P* < 0.01). **D.** Data in *C* regrouped based on gene ontogeny analysis. Microarray studies were performed using the MouseWG-6 whole-genome gene expression array.

We then returned to the 4T1 model to examine effects on spontaneous metastasis. MMP3 expression in 4T1 cells was found to be higher, particularly when compared to CMS4.met.sel cells (Fig. 6A). Silencing MMP3 expression in 4T1 cells (Fig. 6B, based on RNA and protein levels) also significantly reduced tumor growth in *multiple* settings, including: *a)* primary orthotopic growth (Fig. 6C); *b)* experimental lung metastasis (Fig. 6D); *c)* spontaneous lung metastasis even when the primary tumor remained intact, as measured by lung weight or lung nodule count using tissues from mice with similar primary tumor volumes (Fig. 7A); and *d)* spontaneous metastasis following resection of similarly sized primary tumors both 4T1-SC and 4T1-MMP3^lo^ tumor-bearing mice, as measured by prolonged survival (Fig. 7B; Suppl. Fig. 5).

Thus, these data demonstrated for the first time that modulation of tumor-derived MMP3 levels in established tumor cell lines has a profound impact on subsequent stages of the neoplastic process, including progression to metastasis. Changes in MMP3 expression in 4T1 cells, as with the CMS4 system, however, did not alter *in vitro* proliferation or expression of H-2K^d^ (Suppl. Fig. 6).

### Mechanisms by which MMP3 modulates tumor growth and progression

Lastly, we have begun to explore potential mechanisms by which MMP3 expression influenced tumor progression. Here, we focused on whether alterations in MMP3 expression could affect tumor cells in a cell-intrinsic manner. To do so, we first conducted gene expression profiling in the 4T1 model comparing MMP3-expressing vs. MMP3-deficient cells, as described (17). We found that MMP3-deficiency compared to the vector control cells, led to significant alterations in the expression of 58 genes (> 2-fold change, up or down; *P* < 0.01) (Fig. 7C; Suppl. Table 1). Gene ontogeny analyses revealed six major classifications of potential functional relationships, reflecting areas of signaling, immunity/host defense and cell-cell communication (Fig. 7D). All data were deposited in a MIAME-compliant manner and are publically available in the GEO database under accession # GSE68100.

**Figure 8:**
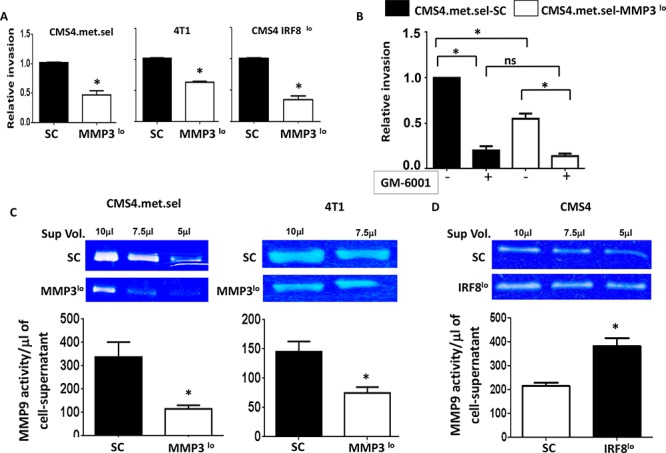
Tumor-derived MMP3 modulates invasive capacity and MMP9 activity *in vitro* **A.** Invasion capacity of the indicated tumor cell line (SC vs. MMP3^lo^), as determined by basement membrane penetration in two-dimensional invasion chamber assays (first bar of each pair set at 1). **B.** Assays in *A* performed in the absence or presence of the pan MMP inhibitor GM-6001 for the CMS4.met.sel model. **C&D.** MMP9 activity, as assessed by zymography. *Upper*, representative gels for the indicated tumor cell line pairs (SC vs. knockdown), which shows *in situ* digestion of gelatin within the gel matrix. *Lower*, quantification for each cell line; data obtained by densitometry of each band. Each value was then divided by the respective sample-volume to yield a measurement of activity per μl of cell-free supernatant. The values for each cell line were then pooled across three separate experiments and reported as mean ± SEM.

Secondly, we investigated the impact of altering MMP3 expression levels on invasive potential. Indeed, we observed a direct correlation between endogenous MMP3 levels and invasive phenotype in all tumor models tested, as measured by *in vitro* invasion assays (Fig. 8A). We then made use of the broad spectrum MMP inhibitor (GM-6001) to verify that invasive ability was MMP-dependent. Using the CMS4-met.sel model, we found that GM-6001 significantly inhibited invasive ability, including the residual activity of the MMP3^lo^ subline (Fig. 8B). One well-known substrate for MMP3 is pro-MMP9 [44], which has been reported to be a potent mediator of tumor progression. Therefore, to determine whether modulation of MMP3 levels in turn affected MMP9 activity, we measured MMP9 activity by zymography. Overall, we found a direct correlation between MMP3 expression and MMP9 activity (Fig. 8C). In both CMS4.met.sel and 4T1 models, the corresponding MMP3^lo^ sublines showed significant declines in MMP9 activity. In contrast, in the CMS4-IRF8^lo^ model, MMP9 activity was significantly elevated compared to the SC control (Fig. 8D). These data indicated that modulation of MMP3 expression impacts tumor progression in a cell-intrinsic manner.

## DISCUSSION

IRF8 was originally characterized as a tumor suppressor gene in certain myeloid leukemias, notably CML [10–12]. Since then, new functional roles for IRF8 have been identified in cancer biology beyond its prototypical link to hematologic malignancies, including its ability to regulate apoptotic death of various types of solid tumors [2, 3, 6, 15, 18–20]. Here, we further advance the notion that IRF8 exhibits antitumor activities, perhaps, distinct from its tumor suppressor functions, and show for the first time that IRF8 acts as a negative regulator of MMP3 expression, a member of the MMP family previously implicated in mammary tumorigenesis and other solid cancers [27].

The importance of IRF8 as a negative regulator of MMP3 expression/function was unveiled using several IRF8 gain- and loss-of function approaches. Knockdown of basal IRF8 levels in tumor cells was accompanied by enhanced MMP3 expression, while the converse held true when IRF8 was overexpressed. Moreover, ChIP assays demonstrated direct binding interactions between the IRF8 protein and ISRE elements of the MMP3 promoter, while luciferase reporter assays revealed a significant inverse relationship between IRF8 expression and MMP3 promoter activity. Interestingly, based on both ChIP and reporter assays, we identified PU.1 as a relevant binding partner of IRF8 consistent with the notion that IRF8 generally does not act alone to modulate transcriptional activity. Future studies are warranted, however, to determine the precise nature of the interaction between these two transcription factors and their association with the MMP3 promoter. The relevance of this IRF8-MMP3 axis in tumor growth *in vivo* was exemplified by the finding that knockdown of MMP3 expression significantly abrogated the enhanced tumor growth effect caused by IRF8-loss. Altogether, these data indicated that IRF8 influenced tumor growth in part through negatively regulating MMP3 expression/function.

MMP3 has been well-recognized as an important player in tumor initiation, strongly based on studies using immortalized epithelial cell lines or transgenic mouse models [27, 29]. Despite its link to early stages of tumorigenesis, the role of MMP3 during later stages of neoplastic progression has remained largely unresolved. Here, we observed a significant positive correlation between MMP3 expression and malignant phenotype and confirmed this observation in multiple tumor models. The causal link between tumor-derived MMP3 expression and malignant behavior was also demonstrated using MMP3 gain- or loss-of-function approaches. The overexpression of MMP3 in the parental CMS4 cell line model, which expressed low basal levels of MMP3, significantly enhanced tumor growth. Conversely, molecular approaches to silence MMP3 expression in more highly aggressive tumor populations which expressed higher basal levels of MMP3 (CMS4.met.sel or 4T1) led to significant declines in primary tumor growth, as well as experimental or spontaneous metastasis. Importantly, using the orthotopic 4T1 model of spontaneous metastasis, we demonstrated that diminution of tumor-derived MMP3 expression significantly prolonged survival in a post-surgical setting. These data attest to the critical importance of MMP3 expression/function as a potential therapeutic target.

Experimental manipulation of MMP3 levels, however, did not result in overt or adverse effects on cellular proliferation or cell-surface expression of several immune-associated molecules, such as MHC class I (e.g., H-2K^d^), as measured *in vitro* in any of the cell lines tested. Although these *in vitro* analyses are not exhaustively comprehensive, they lend support to the notion that the tumorigenic differences observed *in vivo* reflected additional MMP3-driven intrinsic changes within the tumor population, engagement of host-dependent mechanisms or a combination of both factors. The fact that we observed significant changes in MMP9 activity, invasive capacity *in vitro* or, more broadly, the expression of at least 58 genes (by microarray analysis) support the notion that MMP3-dependent mechanisms of tumor progression are complex and intrinsic, but may not necessarily be evident under such *in vitro* conditions. Moreover, the tumor growth patterns seen in wild-type mice were maintained in SCID mice, suggesting that modulation of MMP3 expression did not simply impact tumor growth by subverting adaptive immunity.

Although these data strongly support the notion that modulation of tumor-derived MMP3 expression levels affected tumor progression in a cell-intrinsic manner, this still does not necessarily exclude the possibility for extrinsic mechanisms. To that end, we have begun to examine the tumor microenvironment in both CMS4 and 4T1 models for potential alterations in host-dependent factors, such as angiogenesis and infiltration of tumor-associated macrophages (TAMs). However, preliminary data revealed no significant changes in the magnitude of angiogenesis, as measured by CD31 expression via immunohistochemistry (IHC) or TAM accumulation, as measured by IHC or flow analysis of single cell suspensions of primary tumor tissues (data not shown). However, these initial studies were performed at a single time point using late-stage tumors. Therefore, future detailed studies are warranted to explore these and other potential determinants in a more dynamic and temporal manner during the course of tumor progression. In addition to quantitative changes in TAM frequencies, subsequent studies will explore potential changes in TAM functionality, as well as quantitative/qualitative characteristics of other immune suppressive myeloid cells such as myeloid-derived suppressor cells (MDSCs) or immature/tolerogenic dendritic cells.

In summary, we found that modulation of MMP3 expression/function could significantly alter malignant behavior of established tumor cell line models in part in a cell-intrinsic manner. Moreover, our findings expand our current understanding of how MMP3 is regulated, as well as how IRF8 functions in tumor biology. In this case, we define MMP3 as a novel transcriptional target of IRF8, perhaps, offering a novel molecular explanation for the reported prognostic significance of MMP3 in solid tumor biology [22, 26, 32, 45].

## MATERIALS AND METHODS

### Mice

Female BALB/c mice (BALB/cJ; H-2^d^) and SCID mice (on a BALB/c background) were obtained from the Jackson laboratory (Bar Harbor, ME). All animal studies were approved and performed in accordance with all regulations and requirements established by our institutional animal care and use committee (IACUC).

### Tumor cell lines and plasmids

The parental CMS4 cell line, a chemically induced sarcoma of BALB/c origin [21], was kindly provided by Dr. A. Deleo (University of Pittsburgh, Pittsburgh, PA). The 4T1 cell line, a spontaneously derived mammary carcinoma of BALB/c origin [46] was obtained from ATCC (Manassas, VA). CMS4.met.sel cells were derived from the parental CMS4 cell line as an *in vivo* aggressive variant as previously described [43]. The IRF8-deficient CMS4 cell line (termed, CMS4-IRF8^lo^) was previously established in our laboratory by transfecting CMS4 cells with shRNA targeting IRF8 or a scrambled control (SC) sequence [3]. In separate experiments, the parental CMS4 cell line was transfected with an empty vector control (VC; pcDNA3.1+) or cDNA encoding the full-length mouse MMP3 gene (Origene, Rockville, MD) to generate MMP3-overexpressing CMS4 cells (termed, CMS4-MMP3^hi^), which were maintained and propagated under G418 (Geneticin) selection. In other experiments, parental CMS4, CMS4.met.sel or CMS4-IRF8^lo^ cells were transfected with shRNA targeting MMP3 or a SC sequence (SA Biosciences/Qiagen, Valencia, CA), which were then propagated under Puromycin selection or Puromycin plus Zeocin in the case of the CMS4-SC/CMS4-IRF8^lo^ cell line pair. Similar methods were used to silence MMP3 expression in the 4T1 tumor model. A pcDNA3.1+ expression plasmid encoding the full-length mouse IRF8 gene was used to transfect the 4T1 cell line to generate an IRF8 overexpressing population (termed, 4T1-IRF8^hi^). Transfection of 4T1 cells with an empty vector was used to generate a corresponding control cell line. All cell lines were maintained in RPMI-based medium containing the appropriate selection antibiotic(s).

### Transfection methods

Lipid-based methods were used to stably transfect cells (Lipofectamine 2000; Invitrogen, Carlsbad, CA) according to the manufacturer's protocol. Briefly, ~8 × 10^4^ cells were plated overnight, followed by the addition of 1 μg of cDNA or shRNA admixed with Lipofectamine 2000. Cultures were then incubated for 48 hours, followed by the addition of the appropriate antibiotic for selection. The efficiency of knockdown or overexpression was measured at RNA and/or protein levels, as described below, and stably persisted *in vitro* under appropriate antibiotic selection conditions (Zeocin for shIRF8 plasmid; puromycin for shMMP3 plasmid; G418 for MMP3 over-expression plasmid).

### Tumor growth experiments

Three types of tumor growth studies were performed: subcutaneous (SQ), experimental lung metastasis or spontaneous metastasis to the lung. In the case of the SQ tumor growth model, cells were injected into syngeneic BALB/c mice either in the flank for the CMS4 model (5 × 10^5^ cells per mouse) or into the 4^th^ mammary gland for the 4T1 model (5 × 10^4^ cells per mouse). Tumor growth was measured at 3–4 day intervals, and tumor volumes calculated according to the formula: Volume = (Width^2^ × Length)/2. In the case of the experimental lung metastasis model, the indicated tumor cell line was injected intravenously into the lateral tail vein (2–2.5 × 10^5^ cells per mouse).

In the case of the spontaneous metastasis model, the indicated 4T1 cell population was implanted orthotopically, as above, and the extent of metastasis was then assessed in two distinct groups of mice; that is, the primary tumor remained intact or was surgically removed. In terms of the former setting, when tumor volume approached ethical limits or mice showed signs of morbidity, lungs were removed and analyzed for metastasis histologically. In terms of the latter setting, when primary tumor volume approached 90–120 mm^3^, the primary tumor was surgically resected, and mice followed for ethical endpoints of survival. Mice with primary tumor re-growth were excluded from this analysis.

### Staining lung tissue

India ink was used to visualize lung tumor nodules [47]. Mice were euthanized and the ink solution injected through the trachea. Ink-filled lungs were then retrieved and subsequently fixed using Fekete's solution. Tumor nodules appeared white against an otherwise black background. Nodules were counted under a dissecting microscope.

### Clonogenic assays

Lung tissue from tumor-bearing mice was digested with collagenase (1 mg/ml) plus hyaluronidase (0.1 mg/ml) to form single cell suspensions. Varying dilutions of cell suspensions were then plated in 6-well plates in culture media containing 60 μM 6-thioguanine, as described [48]. The cells were allowed to form colonies over a period of 1–2 weeks based on gross inspection under the microscope. Cells were then fixed with 100% methanol, stained with crystal violet (1%, w/v) and colonies quantified under the microscope.

### Proliferation and viability assays

Assays were performed by ^3^H-thymidine incorporation. Cells were plated at varying concentrations in 96-well plates and then incubated for 24 hours. Cells were pulsed with ^3^H-thymidine (1 μCi per well) for 6 hours and collected using a 96-well cell-harvester, followed by radioactivity quantification using liquid scintillation spectroscopy.

### Flow cytometry

Tumor cells were stained using antibodies directed against H-2K^d^ and then analyzed by flow cytometry using the FACSCalibur (Becton-Dickinson, San Jose, CA). Intracellular staining for IRF8 expression (C-19 antibody; Santa-Cruz, Santa Cruz, CA) was performed, as described [17]. After incubation with the primary antibody, cells were stained with a FITC-conjugated rabbit anti-goat IgG secondary antibody (Jackson Immunoresearch, West Grove, PA). To control for nonspecific IRF8 staining, a matched sample was incubated with an IRF8 blocking peptide (Santa Cruz) for 2 hours prior to staining, as described by the manufacturer. All image analyses were performed using an Image Stream flow cytometer (Amnis, Seattle, WA, USA), as described [49]. Specific IRF8 reactivity was determined by correcting for the difference between the unblocked and blocked samples. Kolmogorov-Smirnov statistics, which is a nonparametric test to measure the difference between two sample distributions, generated ‘D-value’ scores ranging from 0 (i.e., no difference) to 1 (i.e., maximum difference). Following data acquisition, the spatial relationship between IRF8 and nuclear images was also measured using the ‘Similarity’ feature in the IDEAS^®^ software package. The similarity score, a log-transformed Pearson's correlation coefficient between the pixel values of two image pairs, provides a measure of the extent of nuclear localization of IRF8 by calculating the pixel intensity correlation between IRF8 reactivity and DAPI images. Cells with low similarity scores exhibit no correlation between the images (corresponding with a predominant cytoplasmic distribution), whereas cells with high similarity scores exhibit a positive correlation between the images (corresponding with a predominant nuclear distribution). The relative shift in this distribution between two populations (e.g., unblocked vs. blocked) was calculated using the Fisher's Discriminant ratio (‘Rd-value’).

### ELISA

Tumor cells (2 × 10^6^) were plated overnight. After incubation, cell-free supernatants were collected and total secreted MMP3 was measured by Quantikine ELISA kit from R&D Systems (Minneapolis, MN) according to the specified protocol.

### Western blot analysis

The indicated cell lines (3 × 10^6^ cells) were plated overnight. Protein lysates were generated in RIPA buffer (Santa Cruz, Dallas, TX) supplemented with protease inhibitors (100X Halt protease cocktail mix, Thermo Fisher Scientific, Waltham, MA). Total protein was measured by the BCA assay (Thermo Fisher Scientific). Proteins were then separated by 10% gel electrophoresis, followed by transfer to nitrocellulose membrane (Bio-Rad, Hercules, CA). Membranes were incubated sequentially with mouse anti-MMP3 antibody (1:500 dilution; Abbiotech, San Diego, CA) or rabbit anti-IRF8 (Cell Signaling, Danvers, MA) and detected using HRP-conjugated secondary antibody (1:5000 dilution; Promgea, Madison, WI). Bands were visualized by exposure to Chemi-luminescent substrate (Thermo Fisher Scientific). The blot was then stripped and probed again for total actin using anti-mouse anti-actin antibody (1:2000 dilution; Promega).

### Zymography

MMP9 activity was measured by zymography. Briefly, 2 × 10^6^ cells were seeded in 6-well plates and incubated overnight in culture medium. Cell-free supernatants were collected and electrophoresed in 10% polyacrylamide gel containing gelatin (precast gels; Bio-Rad). The gel was then treated successively with renaturation and development buffers (from Bio-Rad), as described [50]. MMP9 activity was revealed by degradation of gelatin within the gel. This was evident by the appearance of a band corresponding to the molecular weight of a protein fragment generated by MMP9-mediated digestion of gelatin. Protein bands were visualized by staining with Coomasie blue 250 (Bio-Rad). Gels were then dried and scanned, and the relevant bands analyzed by densitometry using Image J software (NIH).

### Luciferase assay

Cells were plated at a concentration of 2 × 10^5^/well in 24-well plates and co-transfected with 0.5 μg of mouse MMP3 promoter plasmid (or empty vector) and 100 ng of Renilla plasmid, as described earlier using Lipofectamine 2000. Promoter assays were performed using a luciferase-reporter construct driven by a 1.3 kb fragment of the mouse MMP3, kindly provided by Dr. Andreas Kolb (University of Aberdeen, UK [36, 37]). In the case of transfecting cells with multiple expression plasmids concurrently, cells were incubated with the MMP3 promoter construct, full-length mouse IRF8 cDNA, full-length mouse PU.1 cDNA or the respective empty vector control plasmid (i.e., pcDNA3.1 for IRF8 or pCMV for PU.1). Each DNA component was used at 0.5 μg. In all cases, promoter activity was measured ~24 hours post-transfection using the Dual-luciferase reporter assay system (Promega). Relative activity was calculated by determining the ratio of Firefly to Renilla luciferase.

### Invasion assay

A basement membrane-coated chamber system was used to determine relative motility/invasion capacity (Cytoselect Cell invasion assay kit, Cell Biolabs, San Diego, CA). Tumor cells were cultured under serum-starved conditions from 6–24 hours. Cells were then re-plated in serum-free medium at 3 × 10^5^ cells per well inside the invasion chamber, whereas 10% FBS-containing media was added to the outside well as a chemo-attractant. After 48 hours of incubation, non-invaded cells were wiped from the inside chamber. Cells that had invaded the membrane were stained and quantified according to the manufacturer's protocol. Where indicated, the broad spectrum MMP inhibitor, GM-6001 (25 μM) (EMD Millipore, Billerica, MA) was added at the start of the assay.

### RNA analyses

Total RNA was extracted from cultured cells or freshly harvested tumor tissues by use of a RNA extraction kit (Qiagen, Valencia, CA). One microgram of RNA of each sample was then converted to cDNA by iScript cDNA synthesis kit (Bio-Rad; 50°C for 50 min, 85°C for 5 min). The cDNA was then used for PCR amplification using specific primer sets (initial denaturation at 94°C for 2 min, cycle denaturation 94°C for 30 sec, annealing at 60°C for 30 sec, extension at 72°C for 1 min, repeated for 30 cycles followed by final extension at 72°C for 10 min). PCR products were run on a 2% agarose gel stained with ethidium bromide and visualized under UV light using Bio-Rad Gel-doc system. Quantitative RT-PCR was performed using SYBR-Green (Life-Technologies, Carlsbad, CA) and Bio-Rad DNA engine real-time PCR system. Data were quantified by the ΔΔCt method using the formula-fold change = 2^−ΔΔCt^. All results were then reported as the ratio of the specific mRNA signal normalized to the expression of the housekeeping gene, GAPDH. The primers sets used for the PCR reactions were as follows:

**Table:** 

IRF8 (GenBank:NM_008320)	Fwd 5′CGTGGAAGACGAGGTTACGCTG 3′ Rev 5′ GCTGAATGGTGTGTGTCATAGGC 3′
MMP3 (GenBank:NM_010809)	Fwd 5′ ACTCTACCACTCAGCCCAAGG 3′ Rev 5′ TCCAGAGAGTTAGACTTGGTGG 3′
GAPDH (GenBank:NM_008084)	Fwd 5′ CATCACCATCTTCCAGGAGCG 3′ Rev 5′ ACGGACACATTGGGGGTAGG 3′
β-actin (GenBank:NM_007393)	Fwd 5′ ATTGTTACCAACTGGGACGACATG 3′ Rev 5′ CTTCATGAGGTAGTCTGTCAGGTC 3′

### Histology

4-μm sections were cut from formalin-fixed, paraffin-embedded tumor or lung tissues. Specimens were stained with hematoxylin and eosin (H&E) for histologic analyses. Metastatic lesions were recorded and quantified in a blinded fashion by the pathologist (PNB, a coauthor).

### Chromatin Immunoprecipitation (ChIP) assay

Cells were plated at a concentration of 20 × 10^6^/plate, formalin-fixed (1%) for 10 minutes and then treated with glycine to stop the cross-linking reaction. Cells were lysed using the Santa Cruz ‘cell lysis buffer’ and sonicated (10 pulses each for 10 cycles) in Santa Cruz high salt buffer containing protease inhibitors. DNA concentration was measured after the extraction and reverse cross-linking of sonicated DNA. Sonicated DNA (50 μg) of each preparation was then used for subsequent immunoprecipitation reactions. Accordingly, protein-bound chromatin was immunoprecipitated either using rabbit IgG or the following ChIP-certified antibodies: IRF8 (1:50; Cell Signaling), PU.1 (1:50; Thermo Scientific) or phospho-STAT3 (pY705; 1:50, Cell signaling). Subsequent elution of the antibody-protein complex and reverse cross-linking was performed using the Active Motif CHIP-IT kit (Carlsbad, CA). Eluted genomic DNA was PCR-amplified for using primer sequences surrounding the putative ISRE sequence −1137 bp downstream of the transcriptional start site of mouse MMP3 promoter. To that end, the MMP3 promoter sequence (up to 2 kb from the start site) was obtained from EPD (Eukaryotic Promoter database: http://epd.vital-it.ch/) and then visually inspected for putative ISRE sequences see Suppl. Fig. 1, as reported [51]. Primer pairs flanking the putative ISRE region were as follows:

**Table:** 

−1137 bp	Fwd 5′ AGACCTGTTTTTGAGTGGTCTTT 3′
	Rev 5′ TTTCTCACATTTTCTTTCTGTGC 3′

In addition, the DNA immunoprecipitated with anti-IRF8 or PU.1 antibodies from CMS4 or 4T1-IRF8^hi^ cells was PCR-amplified and subjected to Sanger sequencing (RPCI Genomic Core Facility) to identify the existence of the putative IRF8 binding site. Primer pairs for the PCR and subsequent sequencing reactions, which encompassed a region of 358 bp surrounding the putative binding site, were as follows:

**Table:** 

	Fwd 5′ CACCAAGCACAACCCTTATTC 3′
	Rev 5′ GCTGTCGATGAGAGTCACATTA 3′

### Electrophoretic mobility shift assay

Nuclear lysates was prepared from CMS4-SC cells using the NE-PER kit (Thermo Scientific). Oligonucleotides flanking the putative IRF8 binding site of the MMP3 promoter region were designed reflecting either the wild-type sequence or a mutant version containing insertions at both GGAA and GAAA sites (see below). The corresponding oligonucleotide sequences were then dimerized and labeled with ^32^P (forming a double-stranded phosphorylated probe), followed by incubation for 15 minutes with the cellular lysates. After running the samples onto TBE gels, the gels were dried and exposed to X-ray film overnight to visualize the migration patterns.

**Table:** 

Wild-type	Fwd 5′ GAGAACTCGGAATGGAAATGGATGCC 3′
	Rev 5′ CTCTTGAGCCTTACCTTTACCTACGG 3′
Mutant	Fwd 5′ GAGAACTCGGTGAATGGACTAATGGATGCC 3′
	Rev 5′ CTCTTGAGCCACTTACCTGATTACCTACGG 3′

### Statistical analyses

For comparisons between control and experimental groups, data were expressed as the mean ± SEM for the indicated number of mice or experiments. Statistical analysis was determined using two-tailed unpaired *t*-tests or Log-rank test for survival. *P*-values less than 0.05 were considered significant.

## SUPPLEMENTARY FIGURES AND TABLE


