# Supplementary material for: MMP3-Mediated tumor progression is controlled transcriptionally by a novel IRF8-MMP3 interaction
Source: Oncotarget. 2015 May 8;6(17):15164–79. doi: 10.18632/oncotarget.3897 (PMC4558143; doi:10.18632/oncotarget.3897)
Supplement: Supplementary file 1 [file oncotarget-06-15164-s001.pdf]

SUPPLEMENTARY FIGURES AND TABLE

Mouse MMP3 promoter -1300 to +1

GAATTCTGTCTCCTAAGGATTGCCACCAAGCACAACCCTTATTCTCACACCAGCATAAACAAATATT
CCGCCTTTTTTGTTCAGGGAAGACAAAACACAGATTTTGTTTCATCTAAGGAATAAAAAATTATAG
ACCTGTTTTTGAGTGGTCTTTAAAGAGAACTCGGAATGGAAATGGATGCCTTATTGTGATGTGATG
TTCGGTCTCTGGCCAACTGTCTCTGTCAGGCATTTGCAGTACTGGGGATGTATTCCACAAAAACAGT
AACCCTGATCTTTGTACATCTTCCCAACTGAAAATATAAATTGCACAGAAAGAAAATGTGAGAAAG
AAGAACAAAGAGAGAGAAAATGCAGATCCTATATGTGCACAGCATGTAGTTCACCATAATGTGACT
CTCATCGACAGCATAGTGTGCTCATTAGGAAACAAATGGCTGACAAACAAGCAAATTAAAAGCAC
GCAATGTGTGGTGTTGGGAAGACTGGAGAAGGAGGCTGGAAAGGGGCAAGGGTGGCTAAGTTT
AGCAAAACCTTTTATAAAATGCTTAAGTACTACAGTCATGCATTATACACTGAGAATACAAGTGAGA
CTCTACTGGGGAGGGGAGGGAAGGAGAGGGGAGGGGAGAGAGAGAGGAAAAGAGGTGAGG
CAAATGGAGGGAAGGAGAGAATAGGGGAGGAGTAAAGAAGAGAGAAGAGGAGAAGCAAATT
AAAGTAAAGCATAAGCAAAGAATCTTGGGTGATATTTTTCAACATCAAATCATCTACTACAAAAGA
AAACTTGTGACAAAAATAAAAGATATGAGATAAACCAGGACATTCTAGTTCAGTAAATCATATCAA
TATATGAGTCTTTATAGAAAAAGGTATTATAGGCCCATGATCTTTTAATATATGTGGTCACTGATAGTG
TGGACTGTAGCTATACATGCAGACATTTTCCTTACCTCTTCTCACAGTATTCAGCTTGGGCTTCTGGA
AGTTCTTTGTACAACTTGGACTTTTTACCAAGTTAGTCCACTTCTATCCAAGTCACAAACATTACAGC
TCTGGAAGGACAGTTAAATTTTCCAAAGTGGAAAAAAATGCCCCAGTTTTCTCTTTTGCCAAGGC
AGGAAGCATTTCCTGGAGATTAATCACCATTTGCCTTGCAAAATTAAGAAGGTTTGAAGAACTTAG
TAAAGAAGATTGTATCACCCTACTCTGATTTTTAATTTTTGGAAGTGGTCCCATTTGGATGGAAGCA
ATTATGAGTCAGTTTTCGGGTGACTCTACAAACACAACCACTCTATAAAAGTT

Supplementary Figure S1: Mouse MMP3 promoter region showing location of the putative ISRE region at -1137 relative to the transcription start site (TSS). The first base of the TSS is underlined in a brick red, and the underlined sequence shown in bright red reflects the putative binding motif for IRF8.

Sequencing after PCR amplification of immunoprecipitated DNA:

CMS4 cell line, IP product with IRF8 Antibody
NNNNNNNNNNNNNNNNNCCTTTTTTGNTCNGGGAAGACAAAACACAGATTTTGTTTCATCTAAGGAATAAAAAATTATAGACCTGTTTTT
GAGTGGTCTTTAAAGAGAACTCGGAATGGAAATGGATGCCTTATTGTGATGTGATGTTCGGTCTCTGGCCAACTGTCTCTGTCAGGCATTTGC
AGTACTGGGGATGTATTCCACAAAAACAGTAACCCTGATCTTTGTACATCTTCCCAACTGAAAATATAAATTGCACAGAAAGAAAATGTGAGA
AAGAAGAACAAAGAGAGAGAAAATGCAGATCCTATATGTGCACAGCATGTAGTTCACCATAATGTGACTCTCATCGACAGCA

4T1-IRF8 cell line, IP product with IRF8 Antibody
NNNNANNNNNATTCNNNCTTTTTTGNTCNGGNNNACAAAACACAGATTTTGTTTCATCTAAGGAATAAAAAATTATAGACCTGTTTTTGAG
TGGTCTTTAAAGAGAACTCGGAATGGAAATGGATGCCTTATTGTGATGTGATGTTCGGTCTCTGGCCAACTGTCTCTGTCAGGCATTTGCAGT
ACTGGGGATGTATTCCACAAAAACAGTAACCCTGATCTTTGTACATCTTCCCAACTGAAAATATAAATTGCACAGAAAGAAAATGTGAGAAAG
AAGAACAAAGAGAGAGAAAATGCAGATCCTATATGTGCACAGCATGTAGTTCACCATAATGTGACTCTCATCGACAGCA

CMS4 cell line, IP product with PU.1 Antibody
NNNNNNNNATANTCNNCCTTTTTTNNTCNGGGNNACAAAACACAGATTTTGTTTCATCTAAGGAATAAAAAATTATAGACCTGTTTTTGAG
TGGTCTTTAAAGAGAACTCGGAATGGAAATGGATGCCTTATTGTGATGTGATGTTCGGTCTCTGGCCAACTGTCTCTGTCAGGCATTTGCAGT
ACTGGGGATGTATTCCACAAAAACAGTAACCCTGATCTTTGTACATCTTCCCAACTGAAAATATAAATTGCACAGAAAGAAAATGTGAGAAAG
AAGAACAAAGAGAGAGAAAATGCAGATCCTATATGTGCACAGCATGTAGTTCACCATAATGTGACTCTCATCGACANN

4T1-IRF8 cell line, IP product with PU.1 Antibody
NNNNNANNNNNNNNNNCNNNCTTTTTTGNTCNGGGAAGACAAAACACAGATTTTGTTTCATCTAAGGAATAAAAAATTATAGACCTGTTTT
TGAGTGGTCTTTAAAGAGAACTCGGAATGGAAATGGATGCCTTATTGTGATGTGATGTTCGGTCTCTGGCCAACTGTCTCTGTCAGGCATTTG
CAGTACTGGGGATGTATTCCACAAAAACAGTAACCCTGATCTTTGTACATCTTCCCAACTGAAAATATAAATTGCACAGAAAGAAAATGTGAG
AAAGAAGAACAAAGAGAGAGAAAATGCAGATCCTATATGTGCACAGCATGTAGTTCACCATAATGTGACTCTCATCGACAGCA

Supplementary Figure S2: Sanger sequencing of the DNA immunoprecipitated with anti-IRF8 or PU.1 antibodies, followed by PCR amplification. The presence of the putative binding site in each of the indicated sequences is highlighted in a darker (brick) red.

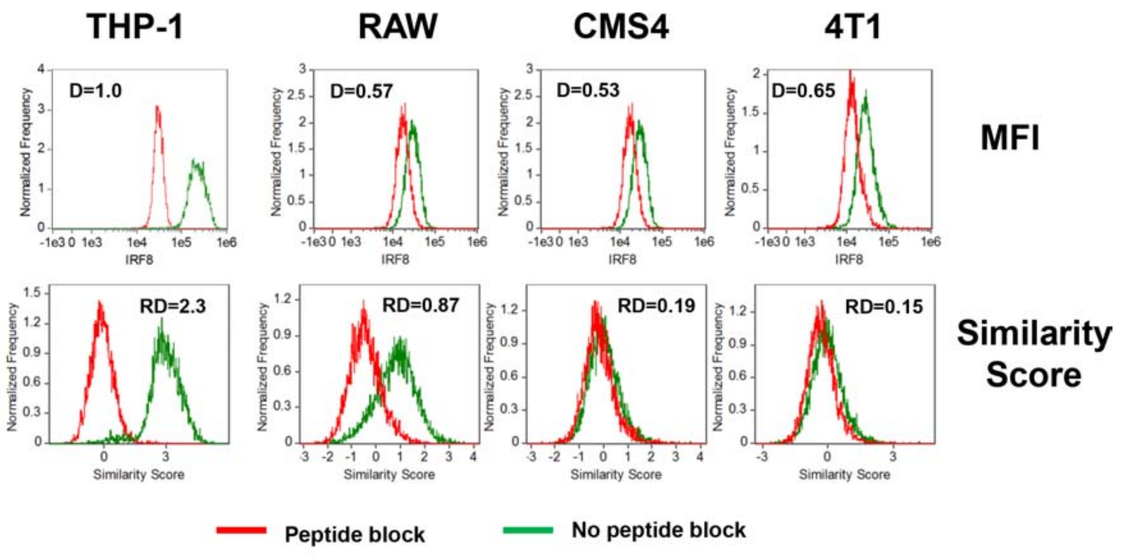

Summary

	$MFI_{NoBlock}$	MFI_{Block}	ΔMFI*	D-value**	Rd-value***
THP1	241,681	31,143	210,538	1.0	2.3
Raw	31,283	18,371	12,912	0.57	0.87
4T1	31,039	14,941	16,098	0.53	0.19
CMS4	34,906	20,088	14,818	0.65	0.15

*ΔMFI: measurement of specific binding of IRF8 antibody
**D-value: measurement of % IRF8 positive cells (1.0 = 100%)
***Rd-value: measurement of nuclear localization of IRF8

Supplementary Figure S3: Image Stream analysis of the distribution of intracellular IRF8 within the cytoplasm or nucleus, as shown in CMS4 or 4T1 cells, in addition to human monocytic THP-1 or mouse macrophage RAW 264.7 cells (top). (Bottom) Summary of data based on D- and Rd-values. Refer to Materials and Methods for experimental details.

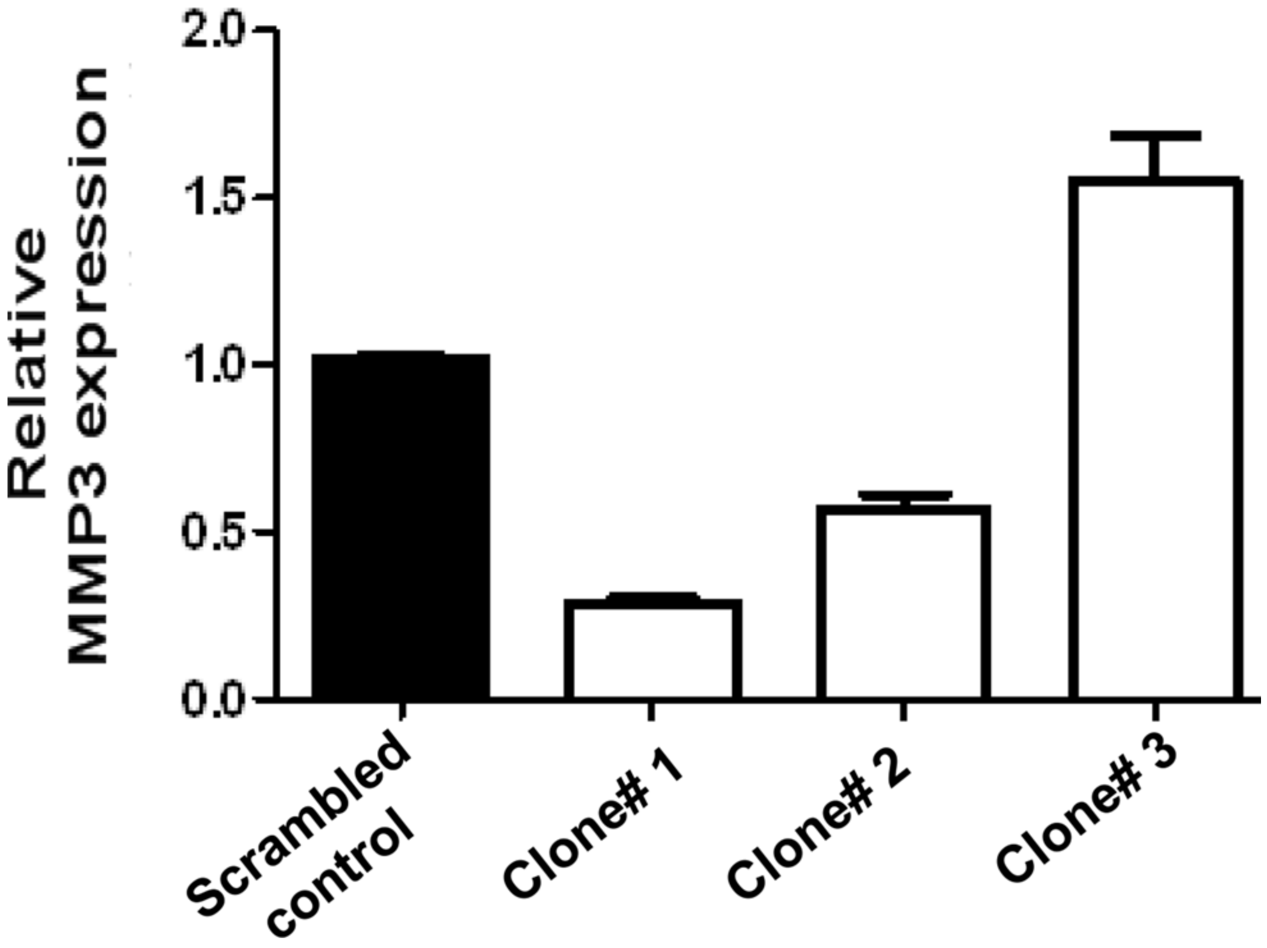

Supplementary Figure S4: Parental CMS4 cells were stably transfected with three separate shRNA-MMP3 constructs or a scrambled control (SC) sequence and the effect of transfection on MMP3 expression was measured by qPCR analyses.

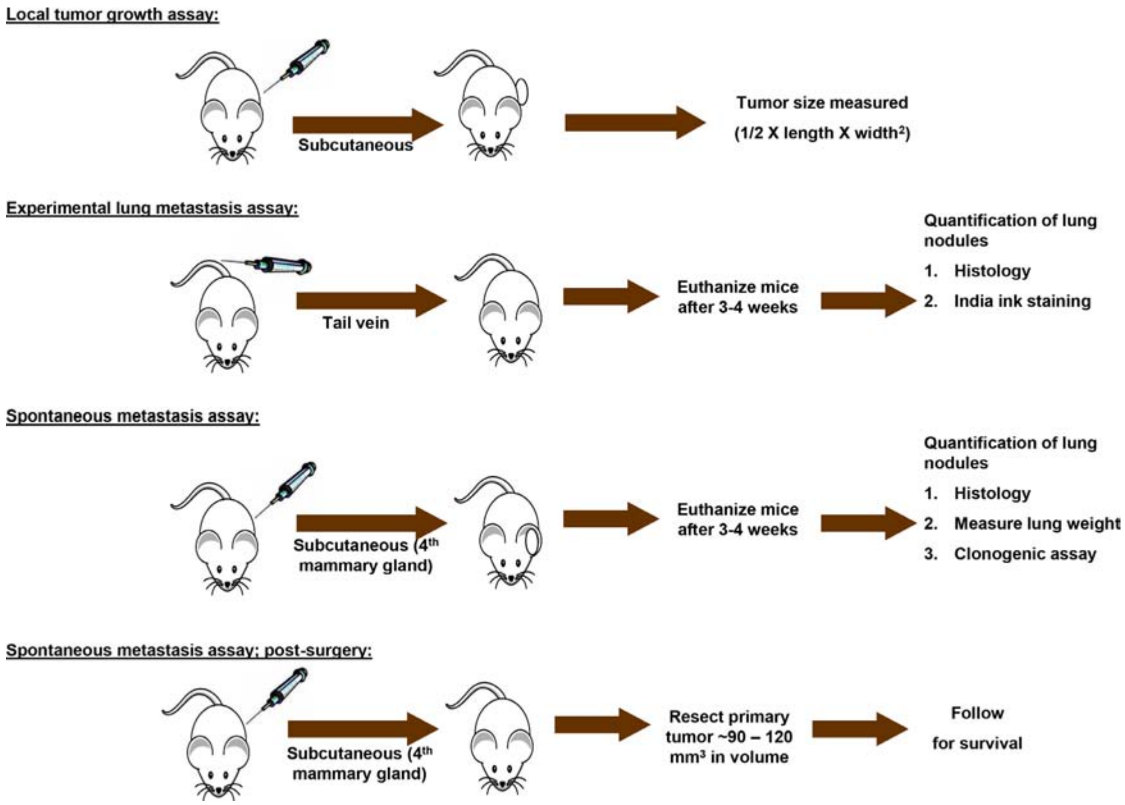

Supplementary Figure S5: Schema reflecting the various *in vivo* tumor growth assays used in this study.

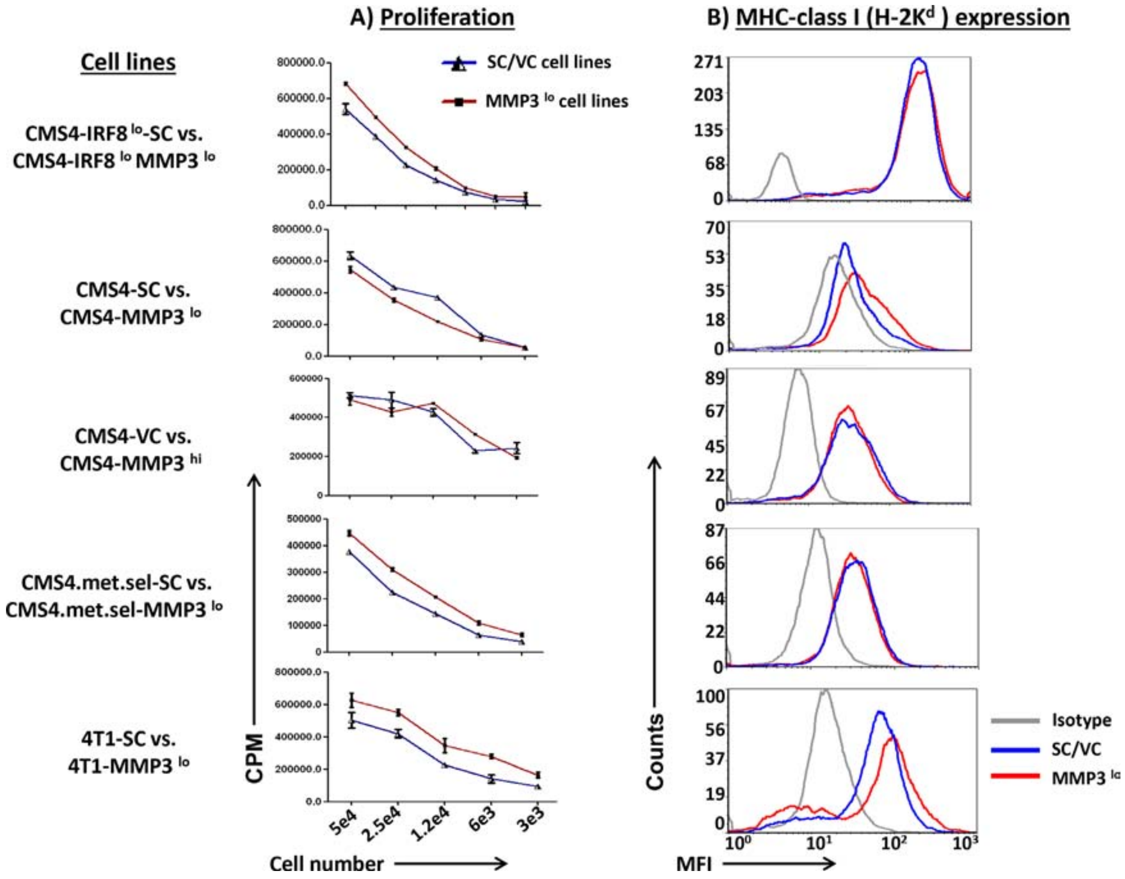

Supplementary Figure S6: Effect of MMP3 knockdown on tumor cell proliferation vitro or expression of MHC class I H-2K^d. A. Indicated tumor cell lines were analyzed for the effect of altering MMP3 and/or IRF8 levels on tumor cell proliferation, as measured by a ^{3}H-thymidine uptake assay. **B.** Cell surface expression of the MHC class I molecule, H-2K^d was analyzed by flow cytometry. Gray line represents staining with the isotype-control antibody.

Supplementary Table S1. Genes Under-or-Overexpressed in 4T1-MMP3[lo] vs. 4T1-SC Cell Populations[a]

Gene Symbol	Fold-change	*P*-Value
Tacstd2	–6.24601	0.000257
Baiap2l1	–4.33928	0.000286
Rbm35a	–3.60367	0.00124
C130090K23Rik	–3.45936	0.003261
Itgb6	–3.44917	0.000207
Inhba	–3.42987	0.001082
Cldn4	–3.39824	0.001095
Rab25	–3.15319	0.004421
Ap1m2	–3.08171	0.000304
Moxd1	–2.9161	0.002954
Lamc2	–2.79129	0.0026
Nuak1	–2.61716	0.000431
Col7a1	–2.55024	0.001098
BC065085	–2.51123	0.004582
Pcyt1b	–2.50124	0.000175
Las1l	–2.48847	0.00073
Rhox5	–2.48442	0.000602
Bnc1	–2.37457	0.000408
Rab32	–2.3601	0.000466
F2rl1	–2.26782	0.00012
Il24	–2.26085	9.80E-07
Tmem54	–2.23405	0.000599
Nox4	–2.18823	0.000177
Sfrp1	–2.17453	0.007577
Fermt1	–2.16986	0.000207
Chst7	–2.06831	0.001582
Mrgprb2	–2.05146	0.002406
Serpina3h	–2.02433	0.001899
Gstm2	2.008708	0.000228
Plscr4	2.014739	0.002958
S100a4	2.021982	5.43E-05
Fah	2.031953	2.29E-05
Mt1	2.033249	0.001043

(Continued)

Gene Symbol	Fold-change	*P*-Value
A530050E01Rik	2.050155	4.43E-05
Otop1	2.050507	6.24E-05
Uap1l1	2.0597	0.002218
Il11ra1	2.071489	9.40E-06
Glipr2	2.146918	0.001204
Cdc42ep3	2.189779	0.001641
Aqp1	2.203865	0.005566
Eno2	2.21411	0.001507
4632417K18Rik	2.235607	0.007009
Sema7a	2.273728	0.007243
Cd74	2.280985	0.000148
Gchfr	2.357398	2.13E-05
Bmp7	2.358297	4.55E-05
Ybx3	2.519784	0.002144
Amigo2	2.526621	5.70E-05
Krt17	2.568909	0.005863
Maged2	2.717067	0.000511
Plekho2	2.777886	0.000131
Dab2	2.791985	0.000334
Ccl5	2.797315	8.96E-06
Olfm1	2.848912	0.000271
Sdpr	2.974933	0.00266
Gpnmb	3.260045	0.002143
Chchd10	3.433784	0.007338
Emp3	3.941261	0.002666

[a]Samples tested in biologic triplicates and results reported as > 2-fold-change (up or down) with *P* values < 0.01. Green and red represent genes under- or overexpressed, respectively.
